# Comparing Short Dental Implants to Standard Dental Implants: Protocol for a Systematic Review

**DOI:** 10.2196/resprot.8836

**Published:** 2018-01-18

**Authors:** Amir Reza Rokn, Abbasali Keshtkar, Abbas Monzavi, Kazem Hashemi, Tahereh Bitaraf

**Affiliations:** ^1^ Dental Implant Research Center Dental Research Institute Tehran University of Medical Sciences Tehran Islamic Republic Of Iran; ^2^ Department of Health Sciences Education Development School of Public Health Tehran University of Medical Sciences Tehran Islamic Republic Of Iran

**Keywords:** dental implants; dental arch; dental restoration failure; postoperative complications/etiology

## Abstract

**Background:**

Short dental implants have been proposed as a simpler, cheaper, and faster alternative for the rehabilitation of atrophic edentulous areas to avoid the disadvantages of surgical techniques for increasing bone volume.

**Objective:**

This review will compare short implants (4 to 8 mm) to standard implants (larger than 8 mm) in edentulous jaws, evaluating on the basis of marginal bone loss (MBL), survival rate, complications, and prosthesis failure.

**Methods:**

We will electronically search for randomized controlled trials comparing short dental implants to standard dental implants in the following databases: PubMed, Web of Science, EMBASE, Scopus, the Cochrane Central Register of Controlled Trials, and ClinicalTrials.gov with English language restrictions. We will manually search the reference lists of relevant reviews and the included articles in this review. The following journals will also be searched: European Journal of Oral Implantology, Clinical Oral Implants Research, and Clinical Implant Dentistry and Related Research. Two reviewers will independently perform the study selection, data extraction and quality assessment (using the Cochrane Collaboration tool) of included studies. All meta-analysis procedures including appropriate effect size combination, sub-group analysis, meta-regression, assessing publication or reporting bias will be performed using Stata (Statacorp, TEXAS) version 12.1.

**Results:**

Short implant effectiveness will be assessed using the mean difference of MBL in terms of weighted mean difference (WMD) and standardized mean difference (SMD) using Cohen’s method. The combined effect size measures in addition to the related 95% confidence intervals will be estimated by a fixed effect model. The heterogeneity of the related effect size will be assessed using a Q Cochrane test and I2 measure. The MBL will be presented by a standardized mean difference with a 95% confidence interval. The survival rate of implants, prostheses failures, and complications will be reported using a risk ratio at 95% confidence interval (*P*<.05).

**Conclusions:**

The present protocol illustrates an appropriate method to perform the systematic review and ensures transparency for the completed review. The results will be published in a peer-reviewed journal and social networks. In addition, an ethics approval is not considered necessary.

**Trial Registration:**

PROSPERO registration number: CRD42016048363; https://www.crd.york.ac.uk/PROSPERO/ display_record.asp?ID=CRD42016048363 (Archived by WebCite at http://www.webcitation.org/6wZ7Fntry)

## Introduction

Dental implants are considered a treatment option to replace missing teeth in edentulous patients. In many clinical situations, insufficient bone volume is a critical limiting factor for dental implant placement and successful osseointegration. Several surgical techniques have been described to obtain adequate bone volume, including bone grafts, sinus lifting, and nerve transposition. These surgeries are technically sensitive and might cause significant postoperative complications such as graft resorptions, severe pain or neurosensory disturbances. Short dental implants have been proposed as a simpler, cheaper, and faster alternative for the rehabilitation of atrophic edentulous areas to avoid the disadvantages of surgical techniques [1-5].

The definition of short dental implants is still controversial in previous research regarding the cut-off length between short and standard implants. Dental implants with intra-bony lengths of less than 10, 8 or 7 mm are defined as short implants in different studies. In this review, implants with lengths of 8 mm or less are considered short because of the available data in research [1,6,7].

Previous systematic reviews have compared short implants with standard implants in the posterior jaws, maxilla or mandible without regards to comparisons between control groups in native or augmented bones [1,7,8]. This comparison may affect outcomes of short implants and two types of control groups with standard lengths [9-12]. Therefore, we not only aim to update existing reviews in more comprehensive databases such as Web of Science, Scopus and clinical trials registries, but also to supplement existing evidence by incorporating the impact of the control group in native or augmented bones.

Our primary objective is to evaluate the marginal bone loss (MBL) of short implants (4 to 8 mm) compared to standard implants (larger than 8 mm) in edentulous jaws. In addition, the survival rate, complications, and prostheses failure of short and standard implants will be assessed as secondary objectives in this review.

## Methods

### Criteria for Considering Studies for This Review

#### Types of Studies

This review will include randomized clinical trials which compared short and standard dental implants in the same study. In these studies, patients were randomized according to a split-mouth or parallel group design to receive short and/or standard implants.

#### Types of Participants

Studies examining patients rehabilitated with short and/or standard dental implants will be included. The patients were 18 years or older and either female or male.

#### Type of Interventions

The intervention of interest is short dental implants of 8 mm or less in length placed in the maxilla and/or mandible.

Comparisons of interest include short dental implants and standard implants.

#### Types of Outcomes

The Primary outcomes will to assess the difference in MBL of short implant (4 to 8 mm) compared to standard implant (larger than 8 mm) in edentulous jaws. In addition, survival rate, complication, and prosthesis failure of short and standard implant will be considered secondary outcomes in the review.

### Search Methods for Identification of Studies

#### Electronic Searches

We will search PubMed, Web of Science, EMBASE, Scopus, the Cochrane Central Register of Controlled Trials, and ClinicalTrials.gov with English language restriction. The following strategy will be used to search PubMed, as listed in Textbox 1.

The PubMed search strategy will be adapted to the syntax and subject headings of the other databases. To complete the electronic search, a manual search in reference lists of relevant reviews (included in this review) in the following journals will be carried out: European Journal of Oral Implantology, Clinical Oral Implants Research, Clinical Implant Dentistry and Related Research.

### Data Collection and Analyses

**Figure 1:**
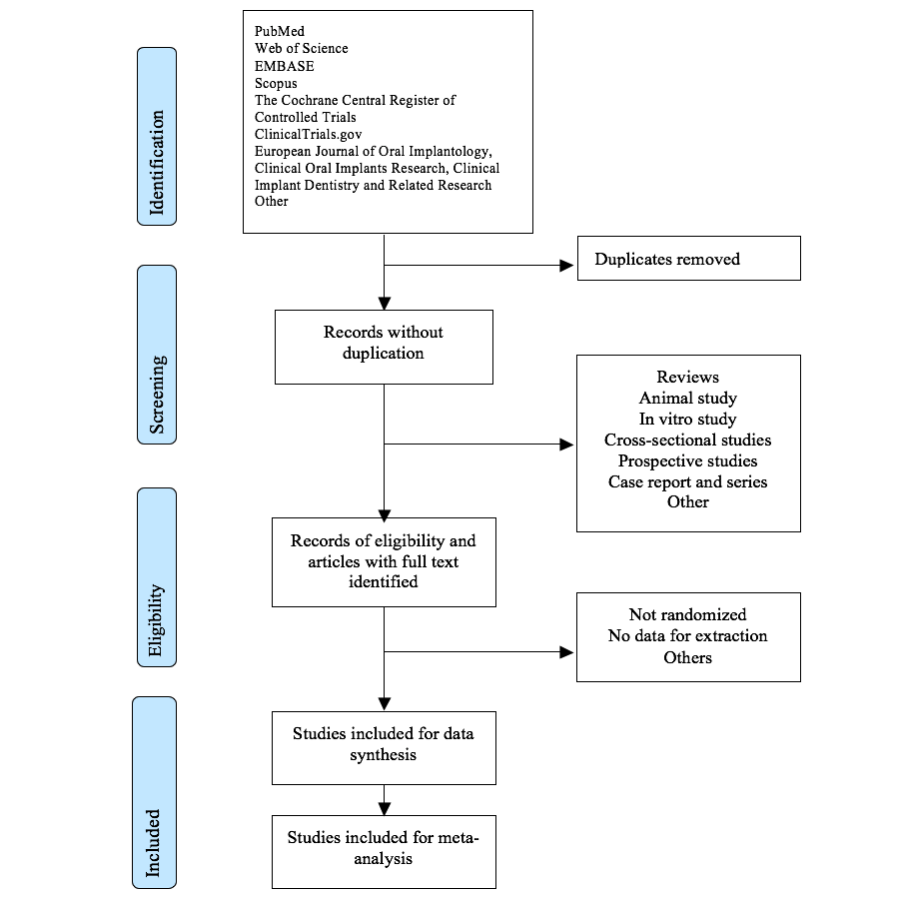
Flow diagram of the study selection process based on PRISMA guidelines.

Two investigators will independently perform the data assessment and extraction using a developed data extraction form. The Preferred Reporting Items for Systematic Reviews and Meta-Analyses (PRISMA) flow diagram shows the study selection process (Figure 1).

The extracted data from each included study will include the following:

1. Study characteristics (author/year of publication, duration of follow up).
2. Short dental implants (number of implants, length and diameter, implant system).
3. Standard dental implants (number of implants, length and diameter, implant system, placement in native or augmented bone).
4. Participant characteristics (number and gender of patients, mean age, number of smokers, arch).
5. Statistics for meta-analysis (mean MBL, implant survival, prosthesis survival, complication).

The main effect size measure in each primary study will be the mean difference between MBL in two arms (groups) after intervention on time intervals. The mean differences will be combined in terms of weighted mean difference (WMD) and standardized mean difference (SMD) by Cohen’s method. The effect size for implant survival will be calculated in terms of risk ratio.

Discrepancies to reach a consensus will be discussed and one arbitrator will adjudicate unresolved disagreements.

**Search methods for the identification of studies.**

**Summary syntax**

#1 (short AND implant) OR “short implant”

#2 Maxilla* OR mandible OR jaws OR “Dental Arches” OR (arch AND dental)

#3 #1 AND #2

#4 (short AND dental AND implant) OR (short AND “dental implant”) OR “short dental implant”

#5 (extra AND short AND implants) OR “extra short implants”

#6 (ultra AND short AND implant) OR “ultra-short implant”

#7 #3 OR #4 OR #5 OR #6

**Complete syntax**

(((short AND implant) OR “short implant”) AND (Maxilla OR mandible OR jaws OR “Dental Arches” OR (arch AND dental))) OR (short AND dental AND implant) OR (short AND “dental implant”) OR “short dental implant” OR (extra AND short AND implants) OR “extra short implants” OR (ultra AND short AND implant) OR “ultra-short implant”

### Assessment of Heterogeneity

The heterogeneity in different effect size measures (WMD, SMD, proportion, etc.) will be assessed by a Q Cochrane test and the related *P* value and I^2^. The I^2^ measures will be classified into mild (between 0% and 25%), moderate (between 25.1% and 50.0%), severe (between 50.1% and 75.0%), and highly severe (between 75.1% and 100.0%). The potential sources of heterogeneity will be found by sub-group analysis or meta-regression methods.

### Risk of Bias in Individual Studies

Two investigators will independently evaluate the methodological quality of included articles according to the Cochrane Collaboration tool for risk of bias [13]. The defined questions will be answered as yes, no, or unclear, and the score of each article will be calculated. Disagreements will be resolved by consensus or consulting a third author.

### Strategy for Data Synthesis

The meta-analyses will be carried out using the STATA version 12 by Mantel–Haenszel and Inverse Variance methods. MBL will be assessed by WMD and SMD with 95% confidence intervals. The survival rate of implants, prostheses failures and complications will be evaluated by a risk ratio with 95% confidence interval. The significance level will be set at *P<*.05 and the statistical tests will be two-tailed.

### Analysis of Subgroups or Subsets

The qualitative data will include: author and publication date; length and number of standard implants in native or reconstructed bone; length and number of short implants; number and gender of patients; mean age; number of smokers; evaluated dental arch; outcomes assessed; follow up duration.

The quantitative data will include: first author, MBL, implants survival, prosthesis survival, and complications.

### Assessment of Reporting Biases

The task of assessing publication or reporting bias will be performed by a funnel plot as well as Begg’s and Egger’s method. If one of the two above-mentioned tests is significant, the Trim and Fill method will be performed to correct the potential reporting bias.

### Sensitivity Analysis

A sensitivity analysis will be used to assess the impact of the outcomes according to the methodological quality items rated by the Cochrane Collaboration tool criteria. Meta-analyses will be performed on high quality studies. The summary table and the review conclusions according to the two meta-analyses will be described. Moreover, the One-Out strategy will be performed by a “metaninf” stata command which is used for assessing impact degree from a specific primary study.

## Results

This protocol of systematic review is aimed at evaluating the MBL of short implants (4 to 8 mm) and standard implants (larger than 8 mm) in edentulous jaws. In addition, the survival rate, complications, and prostheses failure of short and standard implants will be assessed in this review. The outcomes of this review will provide insights on treatment plans that are more preferable and have lower failures and complications. This review is expected to be completed in early-to-mid 2018.

## Discussion

Recently, short dental implants have been proposed as a simpler, cheaper, and faster alternative for the rehabilitation of atrophic edentulous areas to avoid the disadvantages of surgical techniques such as high sensitive technique and postoperative complications. There is no consensus in literature on the performance of short implants compared to standard implants. Some reviews show that MBL, prostheses failures and complication rates of short implants are similar to standard implants. On the other hand, short implants with length less than 8 mm are associated with higher risks of failures due to reduced bone to implant contact [1,5-8].

Other recent systematic reviews were undertaken to compare short implants with standard implants in posterior jaws, maxilla or mandible without comparison between control groups in native or augmented bone [1,7,8]. This protocol updates existing reviews in more comprehensive databases by incorporating the impact of control groups in native or augmented bones.

The primary objective of the study is to evaluate the MBL of short implants (4 to 8 mm) compared to standard implants (larger than 8 mm) in edentulous jaws. In addition, the survival rate, complications, and prosthesis failure of short and standard implants will be assessed as secondary objectives in this review.

## Abbreviations

MBL: marginal bone loss
PRISMA: Preferred Reporting Items for Systematic Reviews and Meta-Analyses
SMD: standardized mean difference
WMD: weighted mean difference
